# The First Neuropalliative Care Unit in Germany—Characteristics of Patients

**DOI:** 10.3390/brainsci12111498

**Published:** 2022-11-04

**Authors:** Anna-Christin Willert, Johanna Meyerling, Christoph J. Ploner, Alexander B. Kowski

**Affiliations:** Department of Neurology, Charité—Universitätsmedizin Berlin, 13353 Berlin, Germany

**Keywords:** neuropalliative care, palliative care needs, palliative care, neurology, brain tumor, neurodegenerative disease, cerebrovascular diseases, palliative care unit, neuropalliative care unit

## Abstract

A unique structure of care for neurological inpatients with significant palliative care (PC) needs was established in the Department of Neurology at the Charité—Universitätsmedizin Berlin in 2021: a specialized neuropalliative care (NPC) unit. After one year, we provide an overview of the concept and the patients’ characteristics. Methods: We retrospectively analyzed the characteristics of patients treated in our NPC unit between February 2021–February 2022. Data were extracted from medical records and PC assessment including diagnosis, mode of admission and discharge, length of stay, and palliative symptoms. Data are presented as averages with a 95% confidence interval [lower limit; upper limit] or percentage (absolute number). Results: We included 143 patients (52% (75) female, 67.9 years [65.6; 70.2]). Patients were admitted from general wards (48%; 68), their homes (22%; 32), intensive care units (16%; 23) or emergency departments (14%; 20). The main diagnoses were tumors of the nervous system (39%; 56), neurodegenerative diseases (30%; 43), neurologic complications (13%; 19) and cerebrovascular diseases (12%; 17). Complaints most frequently rated as severely to overwhelmingly burdensome were motor- or fatigue-associated problems, problems communicating, dysphagia and pain. The average length of stay was 13.7 days [12.2; 15.2]. Forty-five percent (64) of patients were discharged without further PC, 17% (24) were referred to a hospice and 13% (18) were discharged with outpatient PC. Five percent (7) were referred to neurorehabilitation and 21% (30) of patients died. Conclusions: Our NPC unit is a new model of care for neurological patients with substantial PC needs especially within the structures of a highly specialized and individualized medicine.

## 1. Introduction

Patients with incurable neurological diseases have a complex symptom burden on physical, psychosocial, and spiritual levels and may experience impaired autonomy due to limitations in communication, motor skills and cognition. Neurological patients do have a significant need for palliative care [1,2,3,4,5,6,7,8,9]. Approximately 10% of neurological patients have palliative care needs, but only a few of them receive specialized palliative care [10,11,12,13]. Individual and disease-specific illness trajectories, uncertain prognoses especially in neurodegenerative and cerebrovascular diseases, underappreciation of palliative care in neurology and a traditional focus on cancer in palliative care have been identified as major challenges for implementation of neuropalliative care [14].

The need for integration of palliative principles and care structures into the treatment of neurological diseases has however become increasingly recognized [2,11,15,16,17,18]. To capture neurological signs and symptoms, palliative assessment tools were adjusted to [19,20] or validated for neurological patients [21]. Through a specialized palliative care consultation service, it became possible to integrate palliative care in the treatment of neurological inpatients on the general ward, in the stroke unit or intensive care unit [2,11].

Nevertheless, there remains a significant discrepancy between the need for and availability of palliative care structures in Germany, in particular for neurological patients [14,22,23].

To meet the high need of neurological patients for palliative care and to partially cover local demands, in February 2021 the first neuropalliative care unit was established in the Department of Neurology at the Charité—Universitätsmedizin Berlin, Campus Virchow Klinikum, Germany. Starting with five beds, the ward was extended to ten single rooms in October 2021 [24]. The infrastructure was based on structural requirements for palliative care units in Germany. The neuropalliative care unit integrates interdisciplinary neurological and palliative care expertise. Nursing staff mainly trained in palliative care, neurology, and oncology, neurologists with specialist training in palliative care (or those who are currently in training), a psychologist, as well as physio-, speech- and occupational therapists form the multiprofessional and interdisciplinary team. The admission criteria were: (1) uncurable, life-threatening disease of the nervous system with an established diagnosis and a life expectancy of more than a few days, (2) high and complex symptom burden, dynamic worsening of disease course, high treatment effort or undefined goals of care, and (3) neurological symptoms. The palliative care assessment was established according to the recommendation of the German Society for Palliative Care.

In the present report, we provide a detailed characterization of the patients who have been treated in our neuropalliative care unit during the first year of its existence. Our aim was to provide data that may guide the development of concepts of care for neurological patients with significant palliative care needs.

## 2. Materials and Methods

We retrospectively analyzed data of all patients treated in our neuropalliative care unit in the Department of Neurology, the Charité—Universitätsmedizin Berlin, within the first 12 months. Ethical approval was given by the local ethics committee (EA2/234/21). Medical records and palliative care assessment were analyzed for diagnosis, mode of admission and discharge, length of stay, prognosis, disease stage, symptom burden, performance status and existence of a patient decree and healthcare proxy or legal guardian.

A palliative care assessment is routinely performed at the time of admission. It contains standardized tools of self- and third-party assessment to evaluate multidimensional symptom burden. Complaints and symptoms were assessed using the patient-centered “Integrated Palliative Care Outcome Scale for Patients with Long Term Neurological Conditions” (www.pos-pal.org, (accessed on 25 August 2022), IPOS-Neuro V1-P3-10/10/2014 [20,25]). Patients scored 34 symptom-specific items on a 5-point Likert scale from “not at all” (0) to “overwhelmingly” (4) burdensome. IPOS-Neuro was translated into German according to a 6-eyes principle; a validation was waived. In case of severely impaired communication, symptoms were rated by relatives and/or the neuropalliative care team. The disease stage was evaluated as “stable”, “instable”, deteriorating” or “dying”, following the recommendations of the German palliative care guidelines [26]. Prognosis estimation was performed using the “surprise question” (“Would you be surprised if your patient died within the next 12 months?” [21]). Performance status was defined using the performance status scale by the Eastern Cooperative of Oncology Groups (ECOG [27]). ECOG grades the patients self-care ability and physical and daily activity using a 6-point Likert scale from “fully active” (0) to “dead” (5) [27].

Data are presented as averages with a 95% confidence interval [lower limit; upper limit] or absolute number and percentage. Descriptive statistics were performed via SPSS.

## 3. Results

Within the first year, we treated 196 patients; 143 patients were included in our analysis (Table 1). Fifty-three patients were excluded from the analysis because of unanswered or missing palliative care assessments. The total amount of hospitalizations was 218. When patients were re-admitted, only the first stay was included for analysis.

The main groups of diagnoses were neurodegenerative diseases (43; 30%), secondary (35; 24%) and primary (21; 15%) tumors of the nervous system, neurologic complications due to tumors outside the nervous system or tumor-specific therapies (19; 13%) and cerebrovascular diseases (17; 12%) (Table 2).

Symptom burden was rated by patients (69; 48%) or by the care team and/or relatives (70; 49%); in 3% (4) of cases, information about who rated the symptom burden was missing. The complaints most frequently rated as severely to overwhelmingly burdensome were motor problems such as “poor mobility” (93; 65%) and “problems using legs (80; 56%) or arms (57; 40%)”, fatigue-associated complaints such as “fatigue” (77; 54%), “drowsiness” (61; 43%) and “feeling sleepy” (48; 34%) as well as “difficulty communicating” (60; 42%), “problems swallowing” (43; 30%) and “pain” (43; 30%). The average length of stay was 13.7 days [12.2; 15.2].

## 4. Discussion

Our neuropalliative care unit is a new model of care for neurological patients with substantial palliative care needs. Motor and fatigue related complaints, difficulties communicating or swallowing, and pain caused high symptom burden in our patients defined their need for specialized palliative care [11,20]. Autonomy and mobility-affecting symptoms are the main reasons for admission to palliative care wards in Germany [12,13]. Only half of the patients were able to provide ratings of their symptom burden. Neurological symptoms affecting motor, communication and cognitive function made a third-party assessment in many patients necessary, thus emphasizing the indispensability of neurological expertise.

Almost half of the patients had a health care proxy, one fifth had a legal guardian. Patients’ advocates are essential to transmit or rather enforce the patients will while defining goals of care or making therapy decisions. In the light of a missing advance care plan in most patients—only 39% of our patients had a patient decree—this may lead to significant additional caregiver burden. This underlines the high necessity of advance care planning particularly in neurological patients, as deterioration of cognitive and/or motor function may impair communication of patient will in advanced diseases stages [11].

Life expectancy was estimated to be longer than twelve months in almost one-quarter of patients. The proportion of patients dying on our ward (21%) corresponded to experiences with neurological patients receiving palliative care consultation (14–31%) [2,11,28], but the proportion was relatively low compared to other specialized palliative care units in Germany (45–60%) [12,13,29]. Neurological patients especially may have a long disease course with complex and high symptom burden. The proportion of patients (highly) in need of care was less than expected (ECOG 3–4: 60%), whereas almost 90% of neurological patients with palliative care needs who were treated by a palliative care consultation service on our general ward or stroke unit were care-dependent (ECOG 3–4) [11]. To allow an early integration of specialized inpatient palliative care we defined admission criteria that do not exclusively focus on life expectancy and poor functional status but also on high symptom burden and individual disease dynamic.

Surprisingly, after treatment many patients (45%) could be discharged without further specialized palliative care. In particular, patients with neurodegenerative diseases may suffer transient exacerbation of symptom burden resulting in an intermittent rather than a continuous need for palliative care interventions. Thus, the timing, length and continuity of palliative care must be adapted to individual patient needs.

In a small but relevant number of patients (5%), neurological rehabilitation was defined as a palliative treatment objective. Collaboration with well-established neurological, palliative care and rehabilitative structures is therefore still essential.

As most patients in the study were discharged home or to a nursing home, it will be important to provide support to general practitioners, neurologists, and palliative care specialists as home-based primary palliative care providers. This could include interprofessional and interdisciplinary training as well as guidelines for neuropalliative care [14]. To overcome structural challenges, telemedicine may be a suitable approach for some patients [15]. In the long-term, a specialized outpatient neuropalliative care service would be appropriate.

The benefits and problems of the unit must be thoroughly evaluated. Expertise in neurology and palliative care of medical staff (physicians, nurses, etc.) grant appropriate management of communication problems, dysphagia or severe motor impairment. Adjusted admission criteria may unlock paths for neurological patients to access specialized palliative care and allow for early integration. Yet, with subspecialization, standardized criteria must be established for palliative care consultation on a general neurological ward versus treatment on a subspecialized neuropalliative care ward to direct resources. The mean length of stay (14 days) is slightly longer in comparison to other palliative care wards in Germany (10–13 days) [12,13]. Although it is a new component in the neurological patients’ care network, the unit still faces well known bridging problems to outpatient palliative care. In this case as well, the eligibility criteria for hospice care should be defined and assessed. Future research and follow-ups will contribute to a systematic mapping of further benefits and problems.

In our study, we aimed to characterize the group of patients treated in our specialized neuropalliative care unit. Due to the novelty of this structure of care, comparison is difficult: neurological patients on specialized palliative care wards in Germany are underrepresented (1–3%) and the common assessment tools used do not take neurological symptoms into account [12,13]. Limitations result mainly from the single centered, retrospective design: the overall number of patients analyzed is small compared to multicentered studies (7082 patients; [2]) but comparable to other single centered palliative care studies presenting data from a specialized palliative care ward (128 patients; [30]).

Future research is necessary to evaluate the utility of this novel approach for patient- and relative-centered outcomes.

## Figures and Tables

**Table 1:** Patient characteristics and mode of admission/discharge.

143 Patients *	*n* (%)		Advance Care Planning			Mode of Admission	*n* (%)
Age in years	67.9 [65.6; 70.2]		Patient decree	56 (39%)		General ward	68 (48%)
Female	75 (52%)		Health care proxy	79 (55%)		Electively from home/ elderly home	32 (22%)
Male	68 (48%)		Legal guardian	30 (21%)		Intensive care unit	23 (16%)
**Disease Stage**						Emergency department	20 (14%)
Stable	16 (11%)						
Instable	68 (48%)		**12-SQ ^3^**				
Deteriorating	48 (34%)		“Surprised”	33 (23%)			
Dying	2 (1%)		“Not surprised”	95 (66%)		**Mode of Discharge**	
**Functional Status (ECOG) ^2^**						Home/elderly home	64 (45%)
1	12 (8%)					Death	30 (21%)
2	33 (23%)					Hospice	24 (17%)
3	43 (30%)					Home/elderly home with SOPC ^1^	18 (13%)
4	43 (30%)					Rehabilitation	7 (5%)

* Missing information may lead to % sums less than 100%. ^1^ SOPC, specialized outpatient palliative care. ^2^ ECOG [27]: 1 = “restricted in physical strenuous activity”, 2 = “all self-care possible, but unable to carry out work activities, up and about >50% of waking hours”, 3 = “limited self-care possible, confined to bed/chair >50% of waking hours”, 4 = “no self-care possible, permanently confined to bed/chair”. ^3^ 12-SQ [21]: “Would you be surprised if your patient died within the next 12 months?”.

**Table 2:** Spectrum of diagnosis.

*n* = 143		Group of Diagnoses	*n* (%)	Diagnoses	*n* (%)
Neurologic	64 (45%)	Neurodegenerative	43 (30%)	Atypical Parkinsonian disorders	16 (11%)
				Amyotrophic lateral sclerosis	12 (8%)
				Parkinson’s disease	7 (5%)
				Dementia	4 (3%)
				Other	4 (3%)
		Cerebrovascular	17 (12%)	Ischemic	9 (6%)
				Hemorrhagic	4 (3%)
				Other	4 (3%)
		Chronic inflammatory	4 (3%)	Multiple sclerosis	4 (3%)
Neuro-oncologic	75 (52%)	Secondary tumors of the nervous system	35 (24%)	Cerebral metastasis	30 (21%)
				Leptomeningeal cancer	5 (3%)
		Primary tumors of the nervous system	21 (15%)	Glioblastoma	12 (8%)
				Astrocytoma	3 (2%)
				Other	6 (4%)
		Neurological complications due to tumor or tumor-specific therapies	19 (13%)		
**Other**	4 (3%)				

## Data Availability

The raw data supporting the conclusions of this article will be made available by the authors, without undue reservation.
